# Effects of pain on depression, sleep, exercise tolerance, and quality of life in patients with nontuberculous mycobacterial pulmonary disease

**DOI:** 10.1097/MD.0000000000026249

**Published:** 2021-06-11

**Authors:** Kosuke Mori, Mitsuru Tabusadani, Kazumasa Yamane, Satoshi Takao, Yuki Kuroyama, Yusuke Matsumura, Kazuki Ono, Kazuma Kawahara, Shunya Omatsu, Keiji Fujiwara, Koji Furuuchi, Kozo Morimoto, Hiroshi Kimura, Hideaki Senjyu

**Affiliations:** a Department of Clinical Mycobacteriology, Nagasaki University Graduate School of Biomedical Sciences, Nagasaki; b Respiratory Care and Rehabilitation Center, Fukujuji Hospital, Japan Anti-Tuberculosis Association; c Respiratory Diseases Center, Fukujuji Hospital, Japan Anti-Tuberculosis Association, Tokyo; d Department of Basic Mycobacteriology, Nagasaki University Graduate School of Biomedical Sciences, Nagasaki; e Division of Clinical Research, Fukujuji Hospital, Japan Anti-Tuberculosis Association, Tokyo, Japan.

**Keywords:** nontuberculous mycobacterial pulmonary disease, NTM-PD, pain, SF-36

## Abstract

The experience and causes of pain in patients with nontuberculous mycobacterial pulmonary disease (NTM-PD) have not been clarified.

This study aimed to determine the prevalence and severity of bodily pain (BP) in patients with NTM-PD. We also investigated the clinical indicators that contribute to pain.

We used a retrospective cross-sectional study design. The participants were 114 NTM-PD patients (109 women) with a mean age of 65 years. The prevalence and severity of pain were measured using 2 items from the 36-Item Short Form Survey version 2 (SF-36), and the BP score was calculated. Functional limitation due to dyspnea was quantified using the Modified Medical Research Council Dyspnea Scale (mMRC), depression was assessed using the Center for Epidemiological Studies Depression Scale (CES-D), sleep quality was assessed using the Pittsburgh Sleep Quality Index (PSQI); health-related quality of life was assessed using the Leicester Cough Questionnaire (LCQ), and exercise tolerance was measured using the Incremental Shuttle Walk Test (ISWT).

Pain was reported by 70.2% of the patients (n = 80), and of these, 35.7% (n = 25) reported moderate to very severe pain. NTM-PD patients with high levels of pain had significantly higher scores on the mMRC, CES-D, and PSQI scores, and significantly lower performance on the ISWT and LCQ. Multiple regression analysis identified ISWT, CES-D, and PSQI as independent factors that affected BP scores.

Our findings suggest that pain significantly impacts daily life associated with reduced exercise tolerance, the presence of depressive symptoms, and poor sleep quality in patients with NTM-PD.

## 1 Introduction

In recent years, the prevalence of nontuberculous mycobacterial pulmonary disease (NTM-PD) has increased worldwide.^[1,2]^ Furthermore, the prevalence of NTM-PD in Japan is reported to be higher than the global prevalence rates.^[3,4]^ The main symptoms of patients with NTM-PD are chronic cough, phlegm, hemoptysis, malaise, and weight loss, with chronic cough and phlegm being the most common. Furthermore, as the disease progresses, additional symptoms, such as fever and weight loss, arise.^[5–8]^ Munro et al^[9]^ reported that the presence of phlegm has a significant impact on the development of chest pain. Moreover, chronic cough is associated with pain and is thought to result in chest and skeletal muscle pain.^[10–12]^ The assessment of pain is an important element of clinical assessment because the sensation of physical pain leads to inactivity and is thus associated with depression, poor sleep quality, and impaired health-related quality of life (HRQOL).^[13,14]^ Therefore, patients with NTM-PD who typically have chronic cough and phlegm symptoms are at risk of developing pain and may be affected by depression, poor sleep quality, and reduced HRQOL.

Pain associated with respiratory disease has been extensively studied in patients with chronic obstructive pulmonary disease (COPD). In these studies, the prevalence of pain has been reported to be 66% in patients with moderate to severe COPD, and pain intensity was significantly associated with the severity of dyspnea.^[15]^ In patients with NTM-PD, Mehta et al^[16]^ showed that bodily pain (BP) measured using the 36-Item Short Form Survey version 2 (SF-36) was worse than norm-based scores in a Canadian population.

However, there are no previous studies pertaining to the prevalence of pain in patients with NTM-PD, and the experience of pain and its causes in this population are not well understood. The purpose of this study was to determine the prevalence of pain in patients with NTM-PD and to examine the clinical indicators that contribute to pain.

## 2 Materials and methods

### 2.1 Participant enrollment

We used a retrospective cross-sectional study design. The study was conducted at Fukujuji Hospital of the Japan Anti-Tuberculosis Association (Tokyo, Japan), which is a 340-bed facility that specializes in pulmonary diseases and mycobacteriosis in particular. The study was approved by the Institutional Review Board on Human Research of Fukujuji Hospital (approval numbers: 19011, 19020).

From April 2016 to March 2020, 180 patients (160 inpatients) with NTM-PD received pulmonary rehabilitation at Fukujuji Hospital. Pain and exercise tolerance were assessed immediately before the program of pulmonary rehabilitation. In addition, the patients completed several questionnaires. The diagnosis of NTM-PD was made in accordance with the American Thoracic Society/European Respiratory Society/European Society of Clinical Microbiology and Infectious Diseases Society of America criteria by a respiratory physician specializing in infectious diseases.^[17]^ The exclusion criteria were patients whose condition was not stable during the 3 months before assessment as a result of pneumonia or pneumothorax, any recent fractures, those who did not give consent to the study, and patients with missing laboratory data.

### 2.2 Clinical characteristic of the NTM-PD patients

Sex, age, body composition (body mass index [BMI], ideal body weight percent predicted [IBW % pred]), smoking history, duration of disease, presence of any underlying pulmonary disease, and the presence of pain and pain-related medications were recorded from patients’ medical charts. Pulmonary function testing was performed when the patient was in a stable condition, using an electronic spirometer (CHEST AC-8800, Tokyo, Japan) in accordance with published guidelines.^[18]^ Spirometry data comprised forced vital capacity expressed as percent predicted (FVC % pred), forced expiratory volume in one second percent predicted (FEV_1_% pred), and FEV_1_/FVC. Imaging findings obtained from high-resolution computed tomography were classified into the following 4 patterns: noncavitary nodular bronchiectasis type, cavitary nodular bronchiectasis type, fibrous cavity type, and unclassified type.^[19]^ Respiratory symptoms (cough, phlegm, and chest tightness) were assessed using the COPD Assessment Test (CAT).^[20]^ The CAT is an eight-item self-administered questionnaire that includes three items related to respiratory symptoms (cough, phlegm, and chest tightness), with a score of 0 being asymptomatic and 1 to 5 being considered as having respiratory symptoms.^[21]^ Dyspnea was assessed using the Modified Medical Research Council dyspnea scale (mMRC).^[22]^ This scale ranges from 0 to 4, where a higher score indicates greater functional limitations due to dyspnea.

### 2.3 Pain

The SF-36 assesses 36 self-reported health aspects.^[23]^ The questionnaire comprises eight subscales: physical functioning, role-physical, BP, general health, vitality, social functioning, role-emotional, and mental health. All scores were transformed to fit a norm-based score for the general Japanese population, with a mean score of 50 and a standard deviation of 10. Lower scores indicated poorer HRQOL. Two items from the SF-36 were used to measure pain, consistent with a previous study.^[24]^ The first pain question asks, “How much bodily pain have you had during the past 4 weeks?” The response options are “none,” “very mild,” “mild,” “moderate,” “severe,” or “very severe.” The second question asked, “During the past 4 weeks, how much did pain interfere with your normal work (including both work outside the home and housework)?” The choices are “not at all,” “a little bit,” “moderately,” “quite a bit,” or “extremely”. Responses to the first item were used to determine pain prevalence and severity. Responses to the second item were used to determine the impact of pain. We classified patients with BP score <50.0 into the “Pain group” and patients with BP score ≥50.0 into the “No pain group.”

### 2.4 Depressive symptoms

The Japanese version of the Center for Epidemiological Studies Depression Scale (CES-D) was used to screen for depressive symptoms.^[25]^ The CES-D is a 20-item self-administered rating scale developed for the purpose of investigating the prevalence of depressive symptoms in the general population.^[26]^ The highest possible score was 60. A score of 16 or higher indicated the presence of clinical depressive symptoms. As in a previous study,^[27]^ the patients were divided into 2 groups: those who scored <16 were classified as having no depressive symptoms, whereas those who scored ≥16 were classified as having depressive symptoms.

### 2.5 Sleep quality

Sleep quality was assessed using the Japanese version of the Pittsburgh Sleep Quality Index (PSQI).^[28]^ The PSQI generates a 7-component score (range 0–21); the higher the total score, the poorer the subjective sleep quality, and a score >5 is classified as having poor sleep quality.

### 2.6 HRQOL

The Leicester Cough Questionnaire (LCQ) is a symptom-specific quality of life measure for patients with chronic persistent cough.^[29]^ The 19-item questionnaire consists of three domains (physical: 8 questions; psychological: 7 questions; and social: 4 questions). Each item is rated on a 7-point Likert scale for symptoms or the impact of symptoms over the past 2 weeks. The mean score for each of the 3 domains was calculated (range, 1–7). The total score was calculated by summing the domain scores (range 3–21). A higher score indicates a better quality of life. The questionnaire has demonstrated validity and reliability, and is easy to administer and score. Permission to use the Japanese version of the LCQ in this study was granted by Akio Niimi and Haruhiko Ogawa.^[30]^

### 2.7 Exercise tolerance

Exercise tolerance was assessed using the incremental shuttle walk test (ISWT) and conducted in accordance with a standardized protocol.^[31]^ The ISWT is a threshold symptomatic field test that uses a 10-meter course with walking speed dictated by an audio signal. The test is continuous and incremental, with the walking speed increasing every minute. The test was terminated by the patient if they experienced excessive breathlessness or by the operator when the patient failed to maintain the required speed. One practice ISWT was performed to counteract any learning effect. The ISWT distance (ISWD) was recorded and expressed as a percentage of the predicted values obtained from a Japanese sample (ISWD % pred).^[32]^

### 2.8 Statistical analysis

Data are presented as percentages for categorical variables and median (interquartile range) for continuous variables. Data distribution was examined using the Shapiro-Wilk test. For the sensitivity analysis, patients were divided into a “No pain” (BP score ≥50.0) group or “Pain” (BP score <50.0) group. To test for between-group differences in data that conformed to a normal distribution, we used 2-sample *t* tests. The Mann–Whitney test was performed to compare nonparametric data. Categorical variables were analyzed using the*χ*^2^ test.

Associations between BP scores and clinical indicators (age, BMI, FVC % pred, pulmonary symptoms, mMRC grade, ISWD % pred, CES-D, PSQI, and LCQ) were assessed using Pearson or Spearman correlation analyses. In the multiple linear regression analysis, the variables were selected on the basis of *P* value <.2 in the correlation and single linear regression analysis, and forward selection was used to derive the final model. All analyses were performed using SPSS Statistics for Windows version 25.0 (IBM Corp., Armonk, NY). A probability value of *P* < .05 was used to determine statistical significance.

## 3 Results

### 3.1 Clinical characteristics of the study subjects

**Figure 1:**
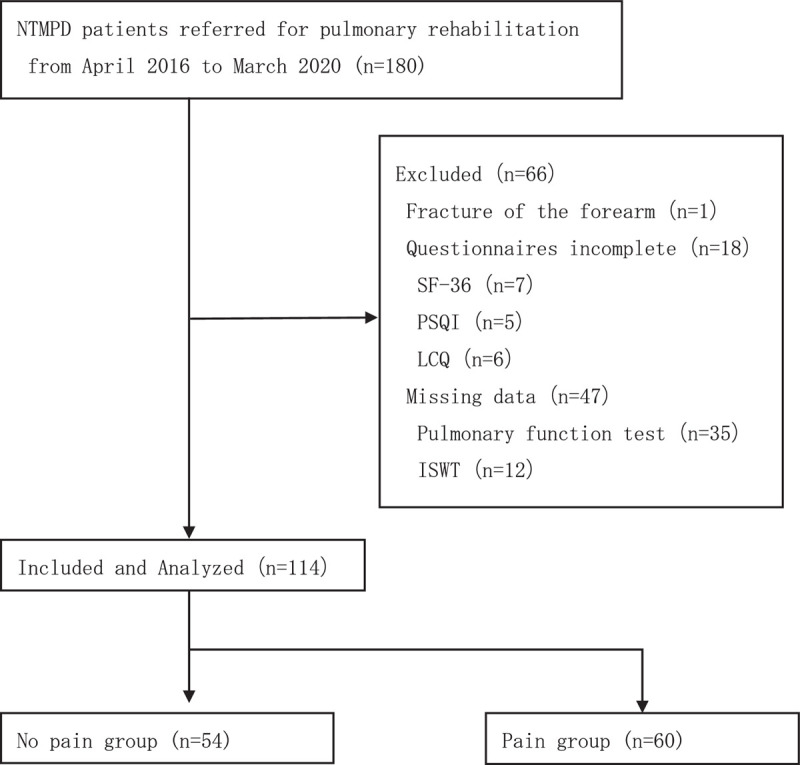
Flow chart of study participants.

During the study period, 1 patient with a history of forearm fractures was excluded. In addition, we excluded 18 patients who did not complete the questionnaires and 47 patients who had missing data. The final sample comprised 114 patients (Fig. 1).

Table 1 presents the clinical characteristics of the study population. The mean age was 65.0 years, and 109 of the sample (95.6%) were women. The median BP score of the sample was 49.0 (interquartile range 40.2–61.4). The number of patients with pulmonary symptoms was 107 (93.9%) for cough, 98 (86.0%) for phlegm, and 78 (68.4%) for chest tightness. The number of patients who were prescribed at least one pain or pain-related medication was 46 (40.4%). Of these, none were prescribed muscle relaxants or opioids. In addition, 41 (36.0%) patients had depressive symptoms, and 64 (56.1%) had poor sleep quality.

**Table 1:** Clinical characteristics of the study subjects (n = 114).

	n/Median	(%)/IQR
Sex (females/males)	109/5	(95.6/4.4)
Age, y	65.0	59.8–71.0
BMI, kg/m^2^	18.2	16.7–20.2
IBW % pred (%)	83	76–92
Smoking history (n)	19	(16.7)
Disease duration, y	4.5	2.0–10.0
Underlying pulmonary disease		
Bronchial asthma	13	(11.4)
Interstitial lung disease	5	(4.4)
Previous tuberculosis	4	(3.5)
COPD	2	(1.8)
Pulmonary function test		
FEV_1_% pred (%)	77	63–92
FVC % pred (%)	79	62–91
FEV_1_/FVC (%)	80	73–85
Radiographic features		
NC-NB	50	(43.9)
C-NB	43	(37.7)
FC	20	(17.5)
Unclassified	1	(0.9)
NTM species		
*M avium* complex	79	(69.3)
*M abscessus* complex	30	(26.3)
Other	5	(4.4)
Respiratory symptoms		
Cough (grade)	2	1–4
Cough (>0)	107	(93.9)
Phlegm (grade)	2	1–3
Phlegm (>0)	98	(86.0)
Chest tightness (grade)	1	0–3
Chest tightness (> 0)	78	(68.4)
Pain and pain-related medications		
Pain medications		
Acetaminophen	17	(14.9)
Nonsteroidal anti-inflammatories	7	(6.1)
Pain-related medications		
Antidepressants	3	(2.6)
Antipsychotics and sleep agents	1	(0.9)
Anxiolytics, sedatives and hypnotics	24	(21.1)
At least one pain or pain-related medication	46	(40.4)
BP (score)	49.0	40.2–61.4
mMRC (grade)	1	0–1
ISWD (m)	440	348–540
ISWD % pred (%)	87	73–105
CES-D (score)	12.0	9.0–18.0
CES-D (>15)	41	(36.0)
PSQI (score)	6.0	4.0–9.0
PSQI (>5)	64	(56.1)
LCQ total (score)	16.3	12.1–19.1

Data are presented as the number (%) of patients, medians (IQR). (>0) in respiratory symptoms indicates some symptoms of cough, phlegm, and chest tightness. BMI = body mass index, BP = bodily pain, CES-D = Center for Epidemiological Studies depression scale, C-NB = cavitary nodular bronchiectasis type, COPD = chronic obstructive pulmonary disease, FC = fibrous cavity type, FEV_1_% pred = forced expiratory volume in one second percent predicted, FVC % pred = forced vital capacity percent predicted, IBW % pred = ideal body weight percent predicted, IQR = interquartile range, ISWD % pred = Incremental Shuttle Walk Test distance percent predicted, ISWD = Incremental Shuttle Walk Test distance, LCQ = Leicester Cough Questionnaire, mMRC = modified Medical Research Council dyspnea scale, NC-NB = noncavitary nodular bronchiectasis type, PSQI = Pittsburgh Sleep Quality Index, VC % pred = vital capacity percent predicted.

**Figure 2:**
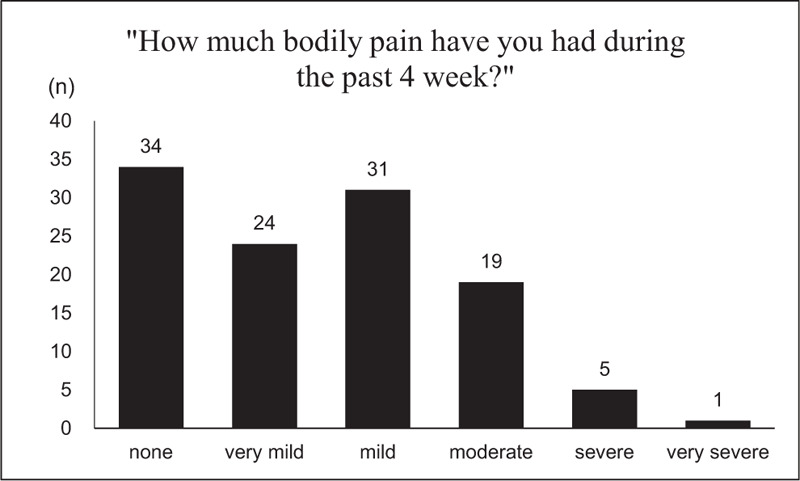
Prevalence and severity of pain. Based on the SF-36 item “bodily pain in the last 4 weeks,” 70.2% of the 114 patients who answered this question reported some pain. A total of 34 patients (29.8%) reported no pain, 24 (21.1%) reported very mild pain, 31 (27.2%) reported mild pain, 19 (16.7%) reported moderate pain, 5 (4.4%) reported severe pain, and 1 (0.9%) reported very severe pain.

**Figure 3:**
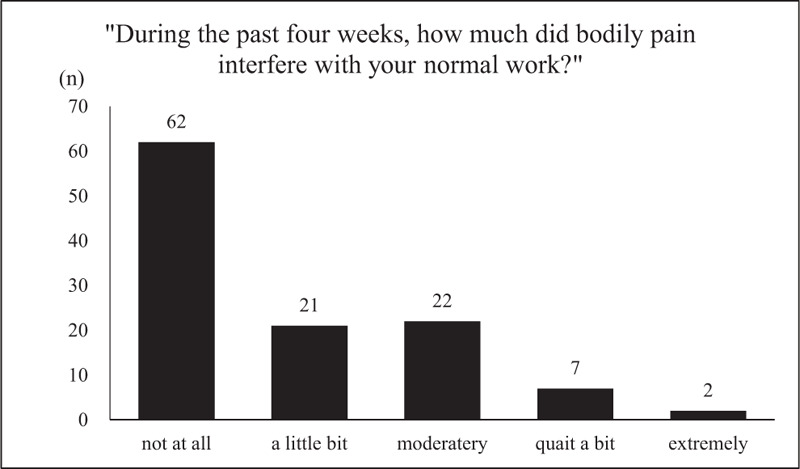
Pain interference with normal work. On the “bodily pain interference with normal work” item, of the 114 patients with NTM-PD who answered this question, 62 patients (54.4%) reported not at all, 21 (18.4%) reported a little bit, 22 (19.3%) reported moderately, 7 (6.1%) reported quite a bit, and 2 (1.8%) reported extremely.

The prevalence and severity of the pain are shown in Figure 2. Pain occurred in 70.2% of patients, with 35.7% reporting moderate to very severe pain. Furthermore, 45.6% of the participants reported that pain interfered with their normal work (Fig. 3).

### 3.2 Comparison of clinical indicators between Pain group and No pain group in patients with NTM-PD

Table 2 shows the characteristics of NTM-PD patients in the pain and no pain groups. The scores for the mMRC (*P* = .007), CES-D (*P* < .001), and PSQI (*P* = .007) were significantly higher, and FVC % pred (*P* = .032), ISWD % pred (*p* = .005), and LCQ total (*P* *=* .001) were significantly lower in the pain group than in the no pain group. Between-group differences were found for pain and pain-related medications (anxiolytics/sedatives/hypnotics [*P* = .014]), and at least 1 pain or pain-related medication (*p* = .009). There were no significant differences in age, sex, duration of disease, smoking history, radiographic features, bacterial species, or respiratory symptoms between the 2 groups.

**Table 2:** Comparison of clinical indicators between Pain group and No pain group in patients with nontuberculous mycobacterial pulmonary disease.

	No pain group (n = 54)		Pain group (n = 60)		
	n/median	(%)/IQR	n/median	(%)/IQR	*P*
Sex (females/males)	52	(96.3)	58	(95.0)	.736
Age, y	64.0	58.8–70.0	68.5	61.3–73.0	.093
BMI, kg/m^2^	18.6	16.5–20.4	17.9	16.7–20.0	.249
IBW % pred (%)	85	75–93	81	76–91	.249
Smoking history (n)	10	(18.5)	9	(15.0)	.615
Disease duration, y	3.5	1.0–9.0	4.8	2.0–11.8	.268
Underlying pulmonary disease					
Bronchial Asthma	5	(9.3)	8	(13.3)	.494
Interstitial lung disease	2	(3.7)	3	(5.0)	.736
Previous tuberculosis	2	(3.7)	2	(3.3)	.915
COPD	1	(1.9)	1	(1.7)	.940
Pulmonary function test					
FEV_1_% pred (%)	80	62–97	75	63–86	.136
FVC % pred (%)	84	59–98	74	65–86	.032
FEV_1_/FVC (%)	79	73–85	80	75–86	.249
Radiographic features					.568
NC-NB	23	(42.6)	27	(45.0)	
C-NB	23	(42.6)	20	(33.3)	
FC	8	(14.8)	12	(20.0)	
Unclassified	0	(0.0)	1	(1.7)	
NTM species					.361
M. avium complex	40	(74.1)	39	(65.0)	
M. abscessus complex	11	(20.4)	19	(31.7)	
Others	3	(5.6)	2	(3.3)	
Respiratory symptoms					
Cough (grade)	2	1–3	2.5	1–4	.064
Phlegm (grade)	2	1–3	2	1–3	.924
Chest tightness (grade)	1	0–2	1	1–3	.066
Pain and pain-related medications					
Pain medications					
Acetaminophen	8	(14.8)	9	(15.0)	.978
Nonsteroidal anti-Inflammatories	1	(1.9)	6	(10.0)	.070
Pain-related medications					
Antidepressants	1	(1.9)	2	(3.3)	.622
Antipsychotics and sleep agents	0	(0.0)	1	(1.7)	.341
Anxiolytics, sedatives and hypnotics	6	(11.1)	18	(30.0)	.014
At least one pain or pain-related medication	15	(28.0)	31	(51.7)	.009
BP (score)	61.4	54.3–61.4	40.2	35.3–44.6	< .001
mMRC (grade)	0	0–1	1	0–1	.007
ISWD (m)	459	378–570	430	296–520	.019
ISWD % pred (%)	93.9	80.0–108.6	81.4	65.2–98.4	.005
CES-D (score)	10.5	7.0–14.0	16.0	11.0–21.0	< .001
PSQI (score)	5.0	3.0–9.0	7.0	5.0–9.0	.007
LCQ total (score)	18.2	15.2–19.9	13.6	11.7–18.2	.001

Data are presented as the number (%) of patients, medians (IQR). BMI = body mass index, BP = bodily pain, CES-D = Center for Epidemiological Studies depression scale, C-NB = cavitary nodular bronchiectasis type, COPD = chronic obstructive pulmonary disease, FC = fibrous cavity type, FEV_1_% pred = forced expiratory volume in one second percent predicted, FVC % pred = forced vital capacity percent predicted, IBW % pred = ideal body weight percent predicted, IQR = interquartile range, ISWD % pred = Incremental Shuttle Walk Test distance percent predicted, ISWD = Incremental Shuttle Walk Test distance, LCQ = Leicester Cough Questionnaire, mMRC = modified Medical Research Council dyspnea scale, NC-NB = noncavitary nodular bronchiectasis type, PSQI = Pittsburgh Sleep Quality Index, VC% pred = vital capacity percent predicted.

### 3.3 Correlations between the BP scores and clinical indicators

Table 3 shows the correlation coefficients between BP score and clinical indicators, and significant correlations were found between BP score and chest tightness symptoms, mMRC, ISWD % pred, CES-D, PSQI, and LCQ total. No significant correlations were found for any of the other indicators.

**Table 3:** Correlations between the Bodily Pain score and clinical indicators.

	*r*	*P*
Age, y	–0.167	.077
BMI, kg/m^2^	0.089	.346
FVC % pred (%)	0.170	.070
Cough (grade)	–0.138	.142
Phlegm (grade)	0.019	.837
Chest tightness (grade)	–0.215	.022
mMRC (grade)	–0.304	.001
ISWD % pred (%)	0.338	<.001
CES-D (score)	–0.481	<.001
PSQI (score)	–0.377	<.001
LCQ total (score)	0.317	.001

Pearson or Spearman correlation coefficients (r) and *P* value. BMI = body mass index, BP = bodily pain, CES-D = Center for Epidemiological Studies depression scale, FVC % pred = forced vital capacity percent predicted, ISWD % pred = Incremental Shuttle Walk Test distance percent predicted, LCQ = Leicester Cough Questionnaire, mMRC = modified Medical Research Council dyspnea scale, PSQI = Pittsburgh Sleep Quality Index.

### 3.4 Factors determining BP score in patients with NTM-PD

Multiple regression analysis was performed to determine the independent factors affecting the BP score (Table 4). Stepwise multiple regression analysis of the clinical indicators identified ISWD % pred, CES-D, and PSQI as independent variables that affected the BP score.

**Table 4:** Factors determining the Bodily Pain score in patients with nontuberculous mycobacterial pulmonary disease.

	Single regression			Multiple regression			
	β	t	*P*	β	t	*P*	Adjusted *R*^2^
Age, y	–0.167	–1.787	.077				0.322
FVC % pred (%)	0.170	1.826	.070				
Cough (grade)	–0.144	–1.545	.125				
Chest tightness (grade)	–0.243	–2.653	.009				
mMRC (grade)	–0.304	–3.381	.001				
ISWD % pred (%)	0.338	3.800	<.001	0.200	2.434	.017	
CES-D (score)	–0.481	–5.802	<.001	–0.356	–4.187	<.001	
PSQI (score)	–0.377	–4.302	<.001	–0.223	–2.664	.009	
LCQ total (score)	0.317	3.542	.001				

Stepwise multiple regression analysis was performed for variables that showed significant associations in correlation analysis and single-line regression analysis. *P* < .05. β = standardized coefficient, BP = bodily pain, CES-D = Center for Epidemiological Studies depression scale, FVC % pred = forced vital capacity percent predicted, ISWD % pred = Incremental Shuttle Walk Test distance percent predicted, LCQ = Leicester Cough Questionnaire, mMRC = modified Medical Research Council dyspnea scale, PSQI = Pittsburgh Sleep Quality Index.

## 4 Discussion

This study is the first to evaluate the prevalence and severity of pain in patients with NTM-PD using a validated pain measure. It is noteworthy that 70% of patients with NTM-PD reported some level of pain, and of these patients, just over one-third reported pain of moderate to very severe range intensity. Moreover, pain interfered with normal work in almost half of our patients.

The BP scores of the subjects in our study were 49.0, which was not different from the norm-based score for the general Japanese population. In previous studies, scores for BP were lower in Canadians with NTM-PD and in a Spanish study of patients with bronchiectasis.^[16,33]^ Conversely, a study of Japanese patients with Mycobacterium avium complex pulmonary disease reported that the BP score did not differ from a norm-based score.^[34]^ In our study, most of the participants were female, and about half reported that pain did not affect their lives, suggesting that the type of work may have affected the BP score.

Previous studies have reported that cough symptoms are associated with pain in the general adult population.^[12]^ We predicted that cough symptoms would also be associated with pain in patients with NTM-PD, whose main symptoms are cough and phlegm. In our sample, many NTM-PD patients had cough symptoms, and although a higher proportion of patients with pain had cough symptoms, this finding was not significant. The lack of studies on pain in NTM-PD makes it difficult to draw definitive conclusions about the cause of pain. Therefore, additional studies using pain-specific measures (eg, Brief Pain Inventory^[35]^ and McGill Pain Questionnaire^[36]^) are needed to clarify the specific etiology of pain reported by NTM-PD patients.

This study showed that pain in patients with NTM-PD was associated with dyspnea, low exercise tolerance, symptoms of depression, poor sleep quality, and impaired HRQOL. The perception of pain and dyspnea is correlated in healthy individuals, and an inverse correlation between pain severity and physical performance has been reported in patients with chronic pain.^[37,38]^ In addition, in patients with COPD, a condition characterized by symptoms of dyspnea and cough, pain is associated with exercise tolerance and physical activity, and pain intensity is associated with dyspnea severity.^[15,39]^ To date, there have been no reports investigating the association between dyspnea, exercise tolerance, and pain in patients with NTM-PD. In our study, NTM-PD patients in the pain group had significantly more chest tightness symptoms, which suggests that dyspnea may have an influence on pain. However, it did not explain the cause or mechanism underlying the pain.

Symptoms of depression, poor sleep quality, and impaired exercise tolerance may be independent predictors of pain in patients with NTM-PD. Pain can result in depression and sleep disturbances, which have been reported to interact with each other.^[40–42]^ Furthermore, in contrast to the findings in patients with depression or pain alone, patients with pain and depression have been reported to experience a greater decrease in physical, mental, and social functioning.^[43]^ In our study, compared with NTM-PD patients without pain, those with pain were significantly more likely to be prescribed anxiolytics/sedatives/ hypnotics, or at least 1 pain or pain-related medication. NTM-PD is a chronic disease that requires prolonged multidrug treatment. During treatment, patients are known to experience negative emotions.^[44]^ In a previous study,^[45]^ patients with NTM-PD showed high levels of depressive symptoms, and cough was found to be an important predictor of the clinical symptoms of depression. Chest physiotherapy, a nonpharmacological intervention, has been reported to reduce the symptoms of cough and phlegm, improve pulmonary function in patients with NTM-PD,^[46]^ and may also help to improve depressive symptoms. However, whether chest physiotherapy improves pain in patients with NTM-PD is unclear and requires further investigation.

This study has several important limitations. First, the sample was almost entirely female, and thus it is not possible to make inferences about males with NTM-PD. Second, because pain assessment was administered as part of the questionnaire, a detailed pain assessment (physical location, type, and frequency of pain) was not undertaken. Therefore, little is known about the causes and characteristics of pain in this sample. Prospective studies are required to enable more detailed assessments. Because of these limitations, our findings cannot be generalized to all patients with NTM-PD.

## 5 Conclusions

This is the first study to investigate the prevalence and severity of pain in patients with NTM-PD using the BP score and the pain domain of the SF-36 to analyze predictors of pain. Approximately 70% of our sample of patients with NTM-PD reported experiencing pain, and of these patients, over one-third reported moderate to very severe pain. Factors predicting pain in patients with NTM-PD included the presence of depressive symptoms, poor sleep quality, and reduced exercise tolerance. Our findings suggest that pain significantly affects the daily life of patients with NTM-PD. All relevant data are within the paper and its Supporting Information files. The funders had no role in study design, data collection and analysis, decision to publish, or preparation of the manuscript.

## Acknowledgments

The authors thank all the subjects, technical staff, management support teams, and colleagues for their cooperation in this study. Dr. Akihiro Ohkado for his statistical advice. We thank Sarina Iwabuchi, PhD, from Edanz Group (https://en-author-services.edanz.com/ac) for editing the draft of this manuscript.

## Author contributions

Hideaki Senjyu was the principal investigator and contributed to the design of the study, administered the funding, supervised the team's work, and made critical revisions to the paper for intellectual content. Kosuke Mori designed the study, drafted, revised, collected, analyzed, and interpreted the data and prepared the paper. Mitsuru Tabusadani contributed to data analysis and made critical revisions to the paper for important intellectual content. Kazumasa Yamane, Satoshi Takao, Yuki Kuroyama, Yusuke Matsumura, Kazuki Ono, Kazuma Kawahara, Shunya Omatsu, Keiji Fujiwara, Koji Furuuchi, Kozo Morimoto, and Hiroshi Kimura collected and interpreted the data, drafted the manuscript, and made critical revisions to the paper for important intellectual content. All authors read and approved the final paper and agreed to be accountable for all aspects of the work.

**Conceptualization:** Kosuke Mori, Hideaki Senjyu.

**Data curation:** Kosuke Mori, Mitsuru Tabusadani, Kazumasa Yamane, Yuki Kuroyama, Yusuke Matsumura, Keiji Fujiwara, Hideaki Senjyu.

**Formal analysis:** Kosuke Mori, Mitsuru Tabusadani, Hideaki Senjyu.

**Funding acquisition:** Hideaki Senjyu.

**Investigation:** Kosuke Mori, Kazumasa Yamane, Satoshi Takao, Yuki Kuroyama, Yusuke Matsumura, Kazuma Kawahara, Kazuki Ono, Shunya Omatsu, Koji Furuuchi, Hideaki Senjyu.

**Methodology:** Kosuke Mori, Mitsuru Tabusadani, Satoshi Takao, Hiroshi Kimura, Hideaki Senjyu.

**Project administration:** Hideaki Senjyu.

**Resources:** Hideaki Senjyu.

**Software:** Hideaki Senjyu.

**Supervision:** Kozo Morimoto, Hideaki Senjyu.

**Validation:** Kosuke Mori, Hideaki Senjyu.

**Visualization:** Kosuke Mori, Hideaki Senjyu.

**Writing – original draft:** Kosuke Mori, Hideaki Senjyu.

**Writing – review & editing:** Hiroshi Kimura.
